# The comparison of pancreatic and hepatic steatosis in healthy liver donor candidates

**DOI:** 10.1038/s41598-021-83871-0

**Published:** 2021-02-24

**Authors:** Bedriye Koyuncu Sokmen, Tolga Sahin, Alihan Oral, Erdem Kocak, Nagihan Inan

**Affiliations:** 1 Department of Radiology, Faculty of Medicine, Demiroglu Bilim University, Büyükdere Street. No: 120 Esentepe – Sisli, 34394 Istanbul, Turkey; 2 Department of Gastroenterology, Demiroglu Bilim University, Istanbul, Turkey; 3 Department of Internal Medicine, Demiroglu Bilim University, Istanbul, Turkey

**Keywords:** Gastroenterology, Medical research, Gastrointestinal diseases

## Abstract

The aim of this study was to investigate the relationship between nonalcoholic fatty liver disease (NAFLD) and pancreatic steatosis (PS) in patients with biopsy-proven NAFLD. 228 patients with biopsy-proven NAFLD patients who admitted to the Faculty of Medicine of Demiroglu Bilim University between 2004 and 2019 were included in the study. Demographic, laboratory, histological and radiological findings of the patients were recorded retrospectively. Hepatosteatosis (HS) levels were measured by both CT and biopsy, while PS levels were measured by 3 different CT-based techniques. 89 (39%) of the patients were female and 139 (61%) were male. The mean body mass index (BMI) was 27.2 ± 4.0. Biochemical parameters were within normal limits. Liver biopsy showed a significant correlation with HS grade on CT scan (p < 0.001). When CT findings were compared, a significant correlation was found between PS and HS (p < 0.05), but there was no correlation between the HS level in biopsy and the pancreatic adiposity on CT (p > 0.05). Our study was the first to compare biopsy-proven NAFLD and PS, and no correlation was found between biopsy-proven NAFLD and PS.

## Introduction

The increasing frequency of obesity is one of the most important health problems in modern medicine. The prevalence of obesity has doubled in the last three decades since the 1980s. A total of 1.9 billion adults were overweight in 2014, and 600 million of these individuals were obese. Currently, obesity and obesity-related disorders such as diabetes mellitus (DM), metabolic syndrome (MetS)) and nonalcoholic fatty liver disease (NAFLD) have become important global health problems^1,2^. MetS was first described by Raevan in 1988 as Syndrome X. It includes obesity, hyperglycaemia, dyslipidaemia, hypertension and insulin resistance (IR). NAFLD is strongly associated with obesity and is also accepted as a hepatic manifestation of metabolic syndrome. NAFLD has become the most common liver disease in developed countries due to increasing obesity, changing eating habits and sedentary lifestyle^3^. Nonalcoholic steatohepatitis (NASH) develops in 30% to 40% of patients with NAFLD. NASH is defined as an advanced, chronic form of NAFLD, and liver cirrhosis can develop in 10–30% of patients with NASH^4^. The increased prevalence of obesity and DM and recent advances in the treatment of chronic viral hepatitis have made NAFLD one of the leading causes of liver cirrhosis worldwide^5^.

The rise in obesity and NAFLD incidence has led to increased attention on ectopic pancreatic fat accumulation, especially in the last two decades. Pancreatic steatosis (PS) was first described by Ogilvie in 1933 in obese individuals. He used ‘pancreatic lipomatosis’ as a term for fatty infiltration in the pancreas^6^. Since this initial description, pancreatic fat deposition has been studied by many researchers in different studies, and many different definitions and nomenclatures have been used, such as pancreatic lipomatosis, lipomatous pseudohypertrophy, nonalcoholic fatty pancreatic disease (NAFPD) and nonalcoholic fatty steatopancreatitis (NASP)^7–9^. Recently, NAFPD has become the most widely used term in the literature for the description of pancreatic fat accumulation.

Pancreatic fat is associated with systemic vascular complications. It is particularly associated with subclinical atherosclerosis and increases the prevalence of diabetic retinopathy^10^. Kim et al. showed that fatty pancreatic infiltration is associated with a higher risk of carotid atherosclerosis in non-obese patients with type 2 DM^11^. Pancreatic steatosis indirect evidence with respect to the relationship acute and chronic pancreatitis. Given the toxic effect of fat on acinar cells, exocrine pancreatic insufficiency could occur in the evolution of pancreatic steatosis, at least theoretically. Fatty infiltration of the pancreas has been proven to be a significant risk factor for pancreatic fistula formation Obesity is a well-known risk factor for PC and there are some preliminary data to support an association between FP and pancreatic cancer too^12^.

In the study by Bi et al., a total of 13 studies involving 49 329 subjects were included. This analyses elucidated the presence of non alcoholic fatty pancreas disease (NAFPD) and was associated with a significant increased risk of metabolic syndrome, hypertension, nonalcoholic fatty liver disease (NAFLD), diabetes mellitus and central obesity. The association between NAFPD and hyperlipidaemia was not statistically significant^13^. As a result, NAFPD is associated with metabolic syndrome.

Many radiological methods have been reported for the evaluation of PS in the literature, such as ultrasound (US), endoscopic ultrasound (EUS), computed tomography (CT) and magnetic resonance imaging (MRI). CT is one of the most commonly used radiologic methods for the evaluation of PS and NAFLD^14^. The power of CT in the evaluation of pancreatic adiposity has been demonstrated in many studies^15^. The efficacy of CT in measuring hepatosteatosis (HS) is more pronounced. CT has become an alternative method for liver biopsy in many transplantation centres due to its strength in the determination of HS in liver transplant donor candidates^16^. Despite the power of CT and many other radiological methods in the diagnosis of HS, liver biopsy is still considered the gold standard method in diagnosing NAFLD^17^. Although many studies have associated NAFLD and NAFPD using different methods, there are no studies comparing NAFPD with biopsy-proven NAFLD. The main aim of this study was to investigate the relationship between NAFPD and NAFLD in patients with biopsy-proven NAFLD.

## Results

A total of 228 patients were included in the study. Of these patients, 89 were female (39%), and 139 were male (61%). The mean age for the study population was 34.3 ± 8.7. The mean height was 169.1 ± 9.6 cm, and the mean weight was 78.0 ± 12.6 kg. The mean BMI was 27.2 ± 4.0. Table 1 shows the general characteristics of the study population.

**Table 1:** Laboratory findings and general demographic characteristics of study population.

	Mean ± SD	Min–max
Age	34.3 ± 8.7	19–57
**Gender**		
Female	89 (39.0)	
Male	139 (61.0)	
Height (cm)	169.1 ± 9.6	140–197
Weight (kg)	78.0 ± 12.6	44–112
BMI (kg/m^2^)	27.2 ± 4.0	18–37.8
Hb (g/dL)	14.4 ± 1.6	9.9–18.1
WBC (10^3^/UL)	7.15 ± 1.91	3.36–12
PLT (10^3^/UL)	248.6 ± 63.4	135–622
INR	1.04 ± 0.09	0.8–1.6
AST (IU/L)	18.5 ± 5.7	10–41
ALT (IU/L)	22.8 ± 14.1	3–103
ALP (IU/L)	72.8 ± 24.4	6–242
GGT (IU/L)	20.8 ± 15.7	3–111
Albumin (g/dL)	4.68 ± 0.32	3.7–5.5
T.biluribin (mg/dL)	0.59 ± 0.31	0.1–2.5
Bun (mg/dL)	12.2 ± 3.1	5–25
Creatinin (mg/dL)	0.79 ± 0.16	0.4–1.3
Na (miliEq/L)	140.1 ± 2.2	135–146
K (miliEq/L)	4.4 ± 0.3	3.5–5.5
FPG (mg/dL)	95.2 ± 9.5	71–168
Insulin (µIU/mL)	11.1 ± 6.2	1.21–45.9
HbA1c (%)	5.3 ± 0.5	2.7–10
HOMA-IR	2.60 ± 1.61	0.25–13.6
T. Cholesterol (mg/dl)	186.0 ± 41.4	90–304
TGL (mg/dl)	118.0 ± 66.1	10–487
TSH (µIU/mL)	2.00 ± 1.21	0.23–8.65
FT3 (pmol/L)	4.83 ± 1.56	1.2–23
FT4 (pmol/L)	13.7 ± 10.3	0.99–138

*BMI* Body mass index, *cm* centimeter, *kg* kilogram, *Hb* Hemoglobin, *WBC* white blood cell, *PLT* platelet, *INR* international normalized ratio, *AST* aspartate aminotransferase, *ALT* alanine aminotransferase, *GGT* gama glutamyl transpeptidase, *ALP* alkaline phosphatase, *Na* sodium, *K* potassium, *FPG* fasting plasma glucose, *HbA1c* Hemoglobin A1c, *HOMA-IR* Insulin resistance, *TGL* tryglyserid, *TSH* thyroid stimulating hormone, *FT3* free T3, *FT4* free T4.

The complete blood count and the other biochemical laboratory findings of the study population were generally within the normal range. Laboratory findings of the study population were correlated with healthy individuals. Table 1 summarizes the average laboratory findings for the study patients.

BMI and PS levels for each CT measurement method were compared. PS was significantly positively correlated with BMI (Table 2). When patients were compared according to HS on CT, PS was significantly correlated with HS according to CT measurements (Table 3).

**Table 2:** The relationship between pancreatic steatosis and BMI in 3 different CT methods.

	BMI				
	No		Yes (> 25)		p*
	n	%	n	%	
**Pancreas mean steatosis grade**					
Grade 1 steatosis	69	88.5	102	68.0	0.003
Grade 2 steatosis	8	10.3	39	26.0	
Grade 3 steatosis	1	1.3	9	6.0	
**P-S steatosis grade**					
Grade 1 steatosis	69	88.5	103	68.7	0.004
Grade 2 steatosis	8	10.3	38	25.3	
Grade 3 steatosis	1	1.3	9	6.0	
**P/S steatosis grade**					
Grade 1 steatosis	69	88.5	103	68.7	0.004
Grade 2 steatosis	8	10.3	38	25.3	
Grade 3 steatosis	1	1.3	9	6.0	

*Spearman correlation.

**Table 3:** Comparison of HS and PS for different CT scan technics.

Liver steatosis on CT			
	No (n = 83)	Yes (n = 145)	p*
Spearman correlation*	1.18 ± 0.41	1.35 ± 0.59	0.025
Spearman correlation*	1.18 ± 0.41	1.35 ± 0.59	0.032
Spearman correlation*	1.18 ± 0.41	1.35 ± 0.59	0.032

*Spearman correlation.

Patients were evaluated according to the degree of pancreatic and hepatic steatosis on CT scans. The degree of pancreatic adiposity was significantly correlated with the percentile degree of HS in the CT scan (Table 4). However, there was no correlation between the level of HS in liver biopsy and the degree of PS on CT scan (p > 0.05) (Table 5).

**Table 4:** Correlation analysis for pancreatic steatosis grade for each CT measument model.

Percentile degree of hepatic steatosis (%)		
	r	p*
Pancreas mean steatosis grade	0.149	0.023
P-S mean steatosis grade	0.142	0.032
P/S mean steatosis grade	0.142	0.032

*Spearman correlation.

**Table 5:** The relationship between HS in biopsy and pancreatic steatosis in CT scan.

	Hepatosteatosis in biopsy				
	No		Yes		p
	n	%	n	%	
**Pancreas mean steatosis grade**					
Grade 1 steatosis	71	74.7	100	75.2	0.692
Grade 2 steatosis	21	22.1	26	19.5	
Grade 3 steatosis	3	3.2	7	5.3	
Grade 1 steatosis	72	75.8	100	75.2	0.732
**P-S mean steatosis grade**					
Grade 2 steatosis	20	21.1	26	19.5	
Grade 3 steatosis	3	3.2	7	5.3	
**P/S mean steatosis grade**					
Grade 1 steatosis	72	75.8	100	75.2	0.732
Grade 2 steatosis	20	21.1	26	19.5	
Grade 3 steatosis	3	3.2	7	5.3	

Spearman correlation analysis was performed for PS in the last step. While BMI was significantly correlated with PS, age and laboratory findings were not correlated with PS in the study population (p > 0.05) (Table 6).

**Table 6:** Correlation analysis between demographic, laboratory data and pancreatic steatosis in study.

Pancreas mean steatosis grade			P-S mean steatosis grade		P/S mean steatosis grade	
	rho	p	rho	p	rho	p
Age	0.127	0.055	0.128	0.053	0.128	0.053
BMI (kg/m^2^)	0.195	0.003	0.200	0.002	0.200	0.002
AST (IU/L)	− 0.010	0.880	0.006	0.931	0.006	0.931
ALT (IU/L)	0.002	0.980	0.009	0.889	0.009	0.889
ALP (IU/L)	0.031	0.641	0.020	0.767	0.020	0.767
GGT (IU/L)	0.082	0.217	0.086	0.197	0.086	0.197
Albumin(g/dL)	− 0.039	0.563	− 0.053	0.430	− 0.053	0.430
T.Bil (mg/dL)	− 0.161	0.065	− 0.155	0.079	− 0.155	0.079
FPG (mg/dL)	0.119	0.074	0.107	0.109	0.107	0.109
Insulin(µIU/mL)	0.124	0.061	0.113	0.088	0.113	0.088
HbA1c (%)	0.021	0.754	0.013	0.848	0.013	0.848
HOMA-IR	0.126	0.057	0.114	0.087	0.114	0.087
Total cholesterol(mg/dL)	0.033	0.623	0.036	0.590	0.036	0.590
TGL (mg/dL)	0.040	0.547	0.035	0.597	0.035	0.597
TSH (µIU/mL)	− 0.053	0.423	− 0.046	0.485	− 0.046	0.485
FT3 (pmol/L)	0.003	0.969	0.004	0.954	0.004	0.954
FT4 (pmol/L)	0.052	0.433	0.051	0.447	0.051	0.447

Spearman correlation analysis.

## Discussion

Many studies have evaluated the relationship between NAFLD and NAFPD by different radiological methods, but no clinical studies have used liver biopsy. Several studies have found a positive correlation between NAFPD and NAFLD using different radiological methods^18–21^, but none of them include liver biopsy, which is considered the gold standard method for the diagnosis of HS. To the best of our knowledge, this is the first study to compare pancreatic adiposity with NAFLD diagnosed by liver biopsy. In our study, although pancreatic and hepatic steatosis rates measured by CT were correlated as in many other studies, no significant correlation was found between liver biopsy findings and PS. In addition, a significant correlation was found between BMI and pancreatic fat levels. The levels of PS were not correlated with the ages or laboratory parameters of the patients in our study. Our study population consisted of only healthy individuals, and we think that the composition of the study population may be the main cause of these results.

Since the pancreas and liver tissues originate from the same endoderm embryologically, ectopic fat accumulation in both organs can be expected. In many studies, the relationship between NAFPD, NAFLD and obesity led many researchers to assume that NAFPD and NAFLD develop due to similar aetiologies. However, recent data show that different molecular mechanisms mediate the pathophysiology and natural history of pancreatic and hepatic steatosis. In addition, liver fat deposition develops through intracellular lipid accumulation in hepatocytes, while fat accumulation in the pancreatic tissue occurs with intercellular adipocyte infiltration in both acinar and islet cells in the interlobular region^22,23^.

The current literature shows that pancreatic tissue is more sensitive to fat infiltration than liver tissue^24^. Although our study population was not composed of obese individuals, a significant positive correlation was found between the degree of pancreatic fat and mean BMI value in our study. We think that this finding reflects both the accuracy and sensitivity of the methods used in our study and confirms the current literature knowledge.

PS has been evaluated in many studies using different radiological methods, such as US, EUS, CT and MRI, and most of these studies are of East Asian origin^10,18,19,25–29^. US can be a simple, inexpensive and widely accessible radiological technique for the detection of PS, but it also has some disadvantages. Pancreatic visualization may not be possible with US in obese patients. Pancreatic fibrosis may be a misleading factor at diagnosis because pancreatic fibrosis shows a hyperechogenic appearance similar to fat accumulation in pancreatic tissue on US. Pancreatic echogenicity is usually compared with liver echogenicity on US, but the liver is a highly metabolically active organ, and its echogenicity can vary continuously. This may lead to different and misleading results in the diagnosis of PS with US^6,30^. The main advantage of EUS is the proximity of the ultrasound probe to the pancreas, which enables higher resolution imaging of pancreatic tissue compared to CT and MRI, but EUS is an invasive procedure and has some disadvantages, such as a high complication risk and sedation requirement. The fact that both transabdominal US and EUS are operator-dependent methods can lead to different outcomes and may cause diagnostic errors^14^. All of these factors make the reliability of studies using US-based methods controversial. To date, many MRI-based radiological techniques have been used to measure PS^27–29^. MRI is a noninvasive and safe radiological method, but different outcomes were obtained in different MRI-based studies in patients with NAFLD and NAFPD. In some MR-based studies, there was a significant positive correlation between HS and PS^31–33^, whereas in some MR-based studies, there was no correlation^34^. Due to these different results in different studies, the power and adequacy of MRI to detect the relationship between NAFLD and NAFPD is controversial. CT is considered one of the most powerful methods in the detection of PS^14,15^.

In many radiological studies, it was found that there was a correlation between the level of pancreatic adiposity measured by CT and histopathological examination of pancreatic tissue^15,35^. In a previous study the corrected value of pancreatic CT attenuation based on splenic attenuation was compared with the histologic pancreatic fat fraction in 62 patients who underwent any type of pancreatic resection. The histologic pancreatic fat fraction was correlated with the P–S (r =  − 0.616, p < 0.01) and P/S (r =  − 0.622, p < 0.01). Based on the above research results, we investigated the relationship between hepatic and pancreatic steatosis parameters measured by non-enhanced CT^15^. CT is also known to be correlated with liver biopsy for the detection of HS in many studies^16^. Since CT is one of the most highly correlated methods with liver and pancreas biopsies, CT was chosen as the reference radiological method in our study.

Radiological studies have shown different degrees of steatosis in different anatomical regions of the pancreas^36^. The development of the pancreas from two separate buds, ventral and dorsal, in the embryological period and differences in this development period have been suggested to be the major cause of this condition^22,36^. In this study, to prevent possible measurement errors due to the non-diffuse distribution of pancreatic adiposity in the parenchyma, the level of pancreatic fat was measured from 3 different anatomical regions of the pancreas using 3 different measurement techniques.

Although pancreatic and hepatic steatosis appear to be similar diseases associated with obesity and MetS, the pathophysiological mechanisms associated with the development of both diseases are different^24^. When studies on NAFPD in the literature are examined, it is seen that the mean BMI of the patient population in our study is similar to that in other studies^13^. Nonetheless, no significant correlation was found between liver biopsy findings and the degree of pancreatic fat on CT. Since the degree of liver steatosis was determined by liver biopsy, which is accepted as the gold standard method, we believe that the data obtained in our study have higher accuracy and reliability than other studies in the literature. The use of liver biopsy as a reference diagnostic method for NAFLD, non-diffuse distribution of pancreatic adiposity, differences in study populations (ethnicity, number of patients), and the use of different radiological methods and measurement techniques may be the main reasons for the different results between our study and other studies. The results of our study showed that although NAFLD and NAFPD appear to be similar diseases, they are not exactly the same disease, do not have the same pathophysiological mechanisms and do not have a simultaneous prognosis.

Our study had some limiting factors, such as the retrospective design and absence of pancreatic biopsy. However, pancreatic biopsy is a highly invasive procedure and does not seem to be useful due to the high risk of complications and ethical concerns. In addition, since ectopic fat accumulation does not show homogeneous distribution in the pancreatic tissue, different and misleading clinical results may be obtained depending on the site of the pancreatic biopsy.

## Conclusion

The relationship between NAFLD and NAFPD is controversial in the literature. Our study is the first to include liver biopsy to study this issue. We concluded that liver biopsy has not been included in the studies so far, and the absence of liver biopsy may be the main cause of different results in studies investigating the relationship between NAFLD and NAFPD. More accurate data can be obtained with studies involving biopsy-proven NAFLD cases.

## Methods

Nine hundred seventy-eight healthy individuals who presented to the Gastroenterology Department of Demiroglu Bilim University Faculty of Medicine as liver donor candidates between January 2004 and January 2019 were reviewed retrospectively. Two hundred sixty-nine live liver donor candidates who underwent liver biopsy for various reasons were identified, and biopsy reports were reviewed retrospectively. Forty-one patients were excluded from the study for various reasons (viral hepatitis B carriers, systemic disease, chronic drug usage, patients with inaccessible CT images, etc.). A total of 228 patients with no systemic disease who underwent liver biopsy were identified and included in the study. The demographic, laboratory and abdominal CT scan findings of the patients were retrospectively reviewed and recorded from the hospital central information system. A CT scan was used for the measurement of both HS and PS. All patients included in the study underwent a non-contrast upper abdomen and contrast triphasic CT imaging protocol for the evaluation of the liver with a 16-detector multidetector computed tomography (MDCT) device (Somatom Sensation—Siemens Medical Systems, Forchheim, Germany).

**Figure 1:**
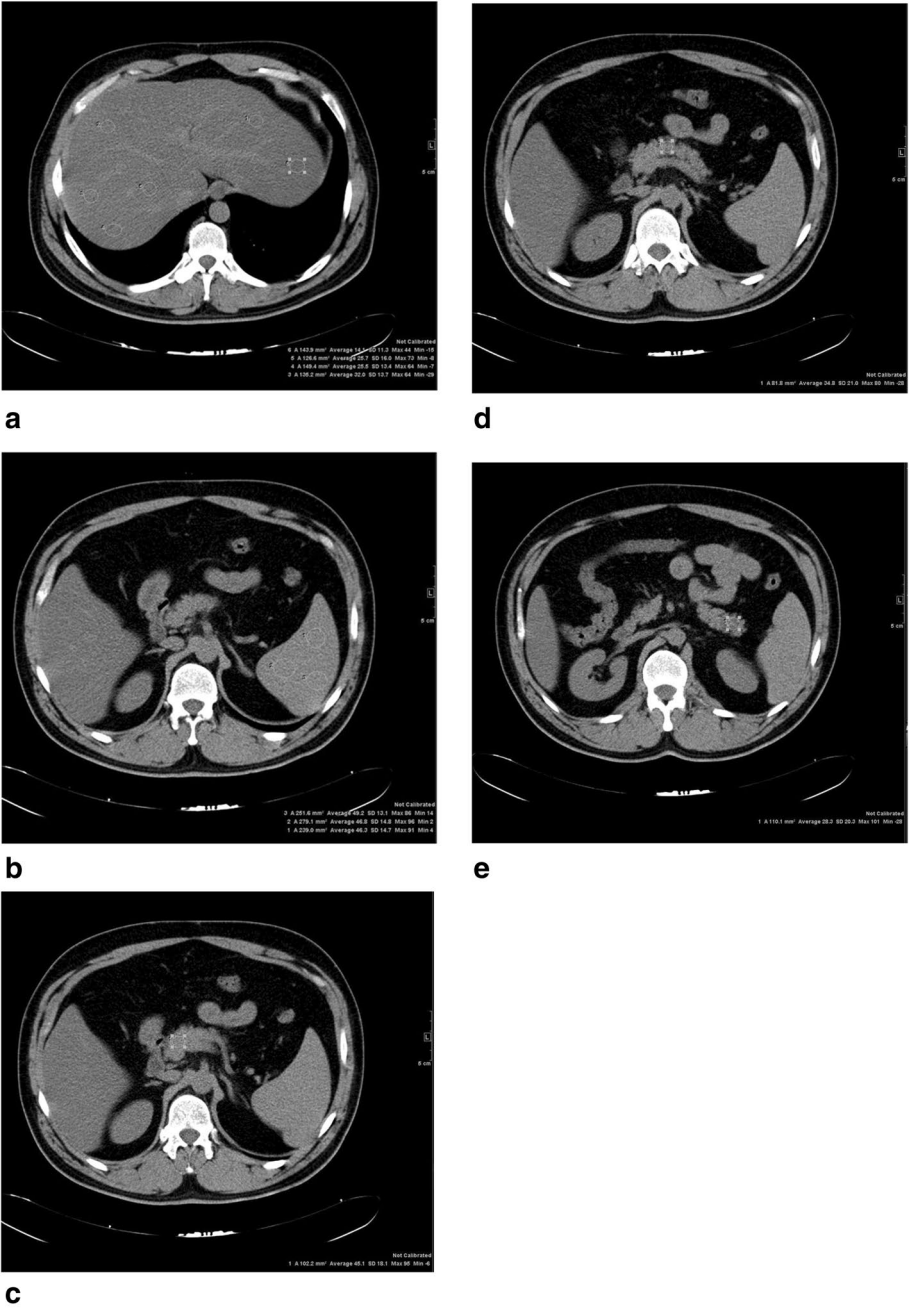
Images of a 35-year-old male who is a candidate for liver donor with hepatostaetosis. (**a**–**e**) Unhanced transverse CT images are shown. HU was measured with ROI from liver, pancreatic head, body, tail and spleen. Mean pancreatic and splenic CT attenuation was 37 and 47 HU, respectively. Difference between pancreatic and splenic attenuation (P-S) and pancreas-to-spleen attenuation ratio (P/S) were − 10 HU and 0.79. Pancreatic steatosis grade 2 was found according to the parameters used. (P_mean_, P-S, P/S). The liver attenuation index was found <  − 10 and was accepted as steatosis > 30%.

The liver attenuation index (LAI) was used to calculate the level of HS in CT. Density measurements were performed on an average of 20 regions of interest (ROIs) in the liver and 10 ROIs in the spleen for the evaluation of HS. Areas away from the vessels were selected for density measurements in both organs. The LAI was calculated by subtracting the mean splenic density from the mean hepatic density. LAI > 5 was accepted as steatosis < 5%, 5 > LAI > − 10 was accepted as steatosis between 5 and 30% and LAI < − 10 was accepted as steatosis > 30%^37^ (Fig. 1).

Non-contrast abdominal CT sections taken with a 16-detector MDCT device (Somatom Sensation—Siemens Medical Systems, Forchheim, Germany) were evaluated for the measurement of PS. For the quantitative assessment of the average fat content of the pancreas, the ROIs were selected from the head, trunk and tail sections of the pancreas, each having an area of approximately 1.0 cm^2^. The Hounsfield Unit (HU) values were measured and averaged from the spleen parenchyma in 3 different regions to normalize PS. Artefacts and vascular structures were excluded in CT images, and the peripheral margin of the pancreas was avoided because of the partial volume effect. Pancreatic mean HU value, HU value difference between pancreas and spleen (P-S) and pancreas and spleen HU value ratio (P/S) were the 3 parameters used to evaluate PS in the study (Fig. 1). Pancreatic fat staging was performed according to these parameters^10,18^. We divided the subjects into three groups according to the degree of each pancreatic steatosis parameter. In our study, our cut-off values for grade 1 pancreatic steatosis were 41.67, − 6.33 and 0.87 for Pmean, P-Smean and P/Smean, respectively. In our study, our cut-off values for grade 2 pancreatic steatosis were 36.67, − 12 and 0.76 for Pmean, P-Smean and P/Smean, respectively. In our study, our cut-off values for grade 3 pancreatic steatosis were 26.5, − 22 and 0.56 for Pmean, P-Smean and P/Smean, respectively. Patients with chronic disease who were taking medication and did not have CT or laboratory findings were excluded from the study.

This study was conducted according to the guidelines laid down in the Declaration of Helsinki, and all procedures involving human subjects were approved by the Demiroglu Science University Ethics Committee (approval number 2019-16-03; approved on 08.06.2019). Informed consent was obtained from all participants.

### Statistical analysis

SPSS 21.0 for Windows was used for statistical analysis. Descriptive statistics: The number and percentage for categorical variables, mean, standard deviation, minimum, maximum for numerical variables were given. The relationship between numerical variables was examined by Spearman Correlation Analysis since no parametric test condition was provided. The Mantel Haenszel chi-square test was used to investigate the relationships between the groups. The agreement of the evaluations was given by the kappa coefficient. Statistical significance was indicated by p < 0.05.
